# A Review of Rickettsial Diseases Other Than Scrub Typhus in India

**DOI:** 10.3390/tropicalmed8050280

**Published:** 2023-05-16

**Authors:** Sivanantham Krishnamoorthi, Shriya Goel, Jasleen Kaur, Kamlesh Bisht, Manisha Biswal

**Affiliations:** 1 Department of Microbiology, All India Institute of Medical Sciences, Bathinda 151001, India; drsivananthamk@gmail.com; 2 Department of Medical Microbiology, Post Graduate Institute of Medical Education and Research, Chandigarh 160012, India

**Keywords:** India, spotted fever group rickettsioses, typhus group rickettsioses, epidemiology, diagnosis

## Abstract

Rickettsial diseases (RD) are widely reported all over the world. Scrub typhus (ST) is a major tropical infection which is well documented all over India. Therefore, the index of suspicion of scrub typhus is high among physicians with regard to patients presenting with acute febrile illness (AFI) and acute undifferentiated febrile illness (AUFI) in India. Rickettsial diseases other than ST (non-ST RDs), which include spotted fever group (SFG) rickettsioses and typhus group (TG) rickettsioses are not uncommon in India, but the index of suspicion is not as high as ST unless there is a history of the presence of fever with rashes and/or recent arthropod bites. This review aims to look into the Indian scenario on the epidemiology of non-ST RDs, especially the SFG and TG rickettsioses based on various investigations, spectrum of clinical presentation, challenges and gaps in knowledge to suspect and diagnose these infections.

## 1. Introduction

Rickettsial disease (RDs) other than scrub typhus (ST) in the Indian context was documented before the centenary by Megaw (1917) in a febrile case of a European male bitten by a tick on his travels from Almora to Lucknow [1]. An outbreak of 12 cases recorded by Major E.S. Phipson (1922) in Shimla due to suspected typhus was investigated and documented using the Weil–Felix reaction with the *Bacillus proteus* X19 strain [2]. Another study from Kashmir in 1951 documented that up to 91% of RDs were due to murine typhus using the complement fixation test (CFT) with other *Rickettsia* spp. potentially implicated in 23% of those cases [3]. Over the last century, scrub typhus was found to be the most prevalent rickettsial disease in India [4,5]. RDs (non-ST RDs) including the spotted fever group (SFG) and typhus group (TG) rickettsioses are not well studied as ST in India. The SFG rickettsioses include Indian tick typhus caused by *Rickettsia conorii*, tick-borne Rocky Mountain spotted fever caused by *Rickettsia rickettsii*, African tick bite fever caused by *Rickettsia africae*, and various tick-borne Rickettsia species, which are responsible for SFG rickettsioses. Additionally, SFG mite-borne Rickettsialpox is caused by *Rickettsia akari.* The TG rickettsioses include louse-borne Epidemic typhus caused by *Rickettsia prowazekii* and flea-borne endemic typhus (murine typhus) caused by *Rickettsia typhi* [6].

Most studies which documented non-ST RDs are based on serological investigations such as the Weil–Felix test (WFT), enzyme-linked immunosorbent assay (ELISA) for IgM and IgG antibodies against *Rickettsia* species such as *Rickettsia conorii* and *Rickettsia typhi*, and indirect immunofluorescent assay (IFA) [6,7,8,9,10,11]. Molecular assay-based studies for non-ST RDs are limited in the Indian scenario. Serological tests such as the Weil–Felix test had been used commonly since the beginning of the twentieth century, and ELISA and IFA are relatively recent additions. Molecular tests have mostly been used for research rather than routine diagnostics in the twenty-first century [12,13,14]. This review focuses on the epidemiology of non-ST RDs based on all of the above tests, particularly the SFG and TG rickettsioses in India to understand the gaps in diagnosis and management of these infections.

## 2. Materials and Methods

A literature search was conducted on studies documenting non-ST RDs focused on SFG and TG rickettsioses in Indian literature. All papers published through July 2022 from PubMed and Embase were retrieved with the terms “India”, “Rickettsia”, “Rickettsial Diseases”, “Spotted fever”, “Typhus Fever”, “Murine Typhus”, “Epidemic Typhus”, “Endemic Typhus”, “Tick Typhus”. In addition, non-indexed papers were retrieved from Google Scholar. Articles focusing only on scrub typhus were excluded, and other members of Rickettsiales (i.e., Anaplasmataceae) were not included in this review. Additionally, review articles and case series were excluded to avoid duplication.

Among the Weil–Felix test-based studies, OX-2 reactivity was considered to indicate SFG rickettsioses, OX-19 reactivity to indicate TG rickettsioses, and weak reactivity to both OX-2 and OX-19 to indicate undifferentiated rickettsioses (OX-K was not considered).

## 3. Results

**Figure 1:**
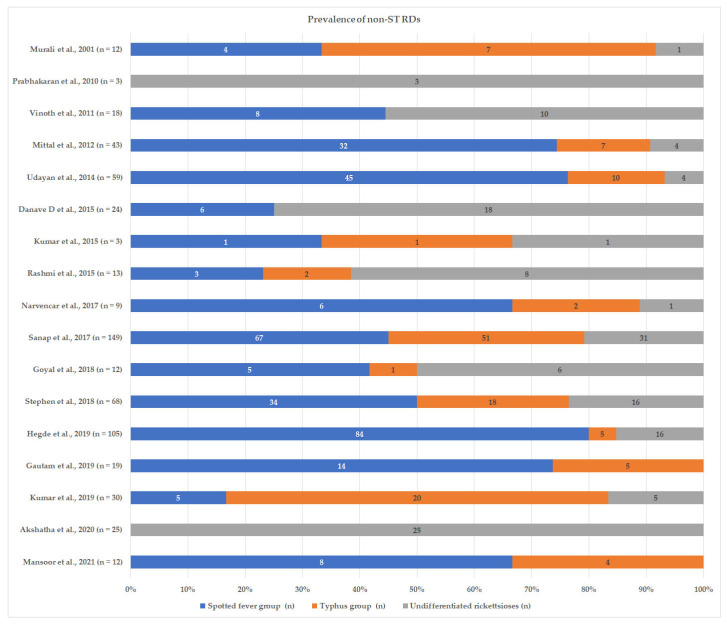
Studies with data on rickettsial diseases other than scrub typhus in India.

Among the documented studies from 1917 to July 2022, there were 16 studies published with data on SFG and/or TG rickettsioses solely using the Weil–Felix test (Table 1), 19 studies using the Weil–Felix test along with other serological tests (Table 2), 10 studies using polymerase chain reaction-based molecular techniques focused on patients (Table 3), and 8 studies (5 out of 8 using molecular techniques) in entomological investigation focusing on associated vectors (Table 4). Other studies included are organ or presentation-specific cases and case series. Studies on SFG rickettsioses outnumber those on TG rickettsioses (Figure 1).

The figures are the number of cases in each rickettsial group among the study population; non-ST RD—Rickettsial disease other than scrub typhus; undifferentiated rickettsioses—low positive titre for SFG as well as TG rickettsioses.

### 3.1. Burden of Non-ST RDs in India

The vast majority of studies on RDs are mostly based on the Weil–Felix test either used as a stand-alone test (Table 1) or in combination with other serological methods [ELISA to detect IgM and/or IgG antibodies against SFG and/or TG pathogens, indirect immunofluorescent assay (IFA), indirect hemagglutination assay (IHA), complement fixation test (CFT), and enzyme-linked dot immunoassay] and a few molecular tests (Table 2 and Table 3). The proportion of SFG rickettsioses in different studies ranges from 0% to 62.5% and that of TG from 1.4% to 88.7% depending on the population included in the studies, selection of test, and setting (outbreak or endemic).

#### 3.1.1. Serological Studies

The Weil–Felix test (WFT) is an agglutination test based on the cross-reactivities of antibodies produced against rickettsiae with certain strains of Proteus vulgaris (OX-19 and OX-2). Though the test is easy and commonly used in India, the sensitivity is low with high specificity [7]. The studies (*n* = 16) from most of the states of India widely used the WFT as the sole diagnostic tool in RDs (Table 1), and the proportion of non-ST RDs among the studied population ranges from 4.6% to 55% [7,18]. The prevalence of SFG and TG rickettsioses ranges from 0% to 45% and 0 to 6.8% respectively [15,16,18,21]. The proportion of samples with undifferentiated rickettsioses with a low positive titre for SFG as well as TG rickettsioses was from 0.5% to 27.6% [17,21]. The prospective studies which used a large number of samples documented the prevalence with a range of 4.6% to 10.1% [7,17,24].

The WFT positive samples were further confirmed for the non-ST RDs using IgM/IgG ELISA and IgM IFA in many studies (*n* = 19) (Table 2). A large community-based cross-sectional survey among people of the northeast states of India using IgG ELISA showed 1.8% of non-SD RDs with 1.4% of SFG and 0.3% of TG rickettsioses [6]. Another community-based cross-sectional survey from people from the southern state of Tamil Nadu showed a prevalence of 15.8% of non-SD RDs with 10.4% and 5.4% of SFG and TG rickettsioses, respectively [30]. A cross-sectional study among AFI patients from Gorakhpur, Uttar Pradesh using IgM/IgG ELISA against *R. conorii* and *R. typhi* showed IgM positivity of 13.6% and 7.1% and IgG positivity of 36.7% and 15.3%, respectively [29].

Among the pyrexia of unknown origin (PUO) or acute febrile illness (AFI) cases irrespective of age group, the non-ST RDs based on IgM antibody ELISA, the prevalence ranged from 1.8% to 26.6% [33,37]. In a study from South India, the prevalence of SFG rickettsioses in adults with acute undifferentiated febrile illness (AUFI) was 1.8% using ELISA [34]. A study showed the prevalence among children with PUO was 1.1% of TG rickettsioses and 7.8% of SFG rickettsioses using enzyme-linked dot immunoassay or micro immunofluorescent assay [32]. The studies targeting children with suspected RDs with defined clinical criteria using ELISA showed SFG rickettsioses of 13.5% in an ICU setting and 32.3% to 37.1% in in-patient settings [10,35,42]. A study among PUO cases using IHA documented 2.6% of non-ST RDs (1.3% SFG and TG rickettsioses each) [31]. A study from Karnataka in a large number of clinically suspected RDs cases using IFA documented 2.1% of SFG and 1.8% of TG rickettsioses. This study also documented cross-reactivity among many *Rickettsia* species (Table 2) [36].

#### 3.1.2. Molecular Studies

Among the limited studies (n = 10, which focused on patients) using molecular tests in India, the burden of non-ST RDs is reported in a wide range from 0% to 59.5% depending on the study population, sample type, and gene targets used (Table 3). The seroprevalence studies involving PCR in samples from asymptomatic subjects and using serum samples or CSF in symptomatic patients with positive serology showed negative to low positivity PCR results [43,44,46,48]. Two studies from northeast Indian states involving random samples from asymptomatic people, residents of ST endemic regions, and serum samples from cases of acute encephalitis syndrome (AES) and PUO resulted in negative PCR results [44,48]. A study investigating a cluster outbreak in three villages of Himachal Pradesh using serology and PCR in seropositive samples were also negative by PCR [43]. Another study investigating the etiology of an AES outbreak from Gorakhpur, using serology and PCR in serum and/or CSF samples resulted in low (1%) positivity [46]. This is not surprising, as PCR positivity is reported to be maximum before 10 days while antibody detection peaks in the second and third weeks [56].

PCR-based studies involving clinically defined cases and/or defined sample type showed a higher positivity [12,13,45,47,49,50]. Two studies from a single center in north India used clot samples from AUFI patients. One of the two studies showed 7% rickettsial diseases, of which 4% were due to SFG and 3% were due to TG. Another study from the same center documented 5.8% of SFG among the investigated study population using SFG-specific gene targets. [13,49]. The most definitive study using stringent criteria, which is recommended by Fournier et al. [57] for the diagnosis of rickettsial species, was carried out by Prakash et al. [12]. A skin biopsy from patients with fever and rash was used as a sample and reported a 58.6% positivity for SFG rickettsioses [12]. Another study from the same center reported 22% of SFG rickettsioses using PCR in clinically defined cases of fever with rashes and 28% by IgM ELISA [50]. One study detected a novel *Rickettsia* species closely related to *Candidatus* Rickettsia kellyi [45]. Another study from south India using whole blood samples from patients with AFI cases documented 59.5% PCR positivity from seropositive samples [47].

#### 3.1.3. Co-Infections

Most of the studies on RDs are from PUO or AUFI where the commoner causes of fever were excluded. Very few studies documented all the causes of fever among patients with acute febrile illness (AFI). A study showed 27% of acute febrile patients were positive for SFG rickettsioses as well as either of other infections such as typhoid, malaria, dengue, and hepatitis [37]. Another study reported 4.6% of RDs with other infections such as dengue, hepatitis, enteric fever, leptospirosis, Lyme disease, measles, and Japanese encephalitis (JE) [27].

#### 3.1.4. Pregnancy

There are no studies focusing particularly on AFI in pregnancy. One study found 35.7% of patients of non-ST RDs (SFG—21.4% and TG—14.3%) in the study population were pregnant or in the peripartum period. The infections were transient and responded to commonly used antibiotics [13].

#### 3.1.5. Travel-Related Non-ST RDs

In 2008, a fatal case of *R. conorii* subsp. *Israelensis* was diagnosed using the molecular method/PCR on an Indian traveler in Israel [58]. Another case of SFG rickettsiosis /typhoid co-infection was reported in Israel from an Indian traveler in 2018 [59]. A case of novel SFG *Candidatus* R. indica Tenjiku01 was reported in Japan from a traveler returning from Karnataka [60]. Two other possible cases of RDs (African tick-bite fever) were reported in Germany and Massachusetts, from cases with a history of travel to India and Africa [61,62]. A traveler who returned from India to the United Kingdom and another who returned to Boston after a short stay in urban Mumbai were reported to have murine typhus [63,64].

#### 3.1.6. Role of Climate and Ecology

RDs were mostly documented to occur in rainy cooler months (July to December) and few studies documented the cases in spring to summer months [3,13,31,34,35,37,49]. The tick *Rhipicephalus sanguineus*, an important vector of the Indian tick typhus pathogen *R. conorii* is widely distributed all over India (Andhra Pradesh, Arunachal Pradesh, Assam, Bihar, Chhattisgarh, Gujarat, Haryana, Himachal Pradesh, Jammu and Kashmir, Jharkhand, Karnataka, Kerala, Madhya Pradesh, Maharashtra, Mizoram, Nagaland, Odisha, Punjab, Rajasthan, Tamil Nadu, Uttar Pradesh, West Bengal) [65].

Various studies in different parts of India have looked for the presence of rickettsial pathogens in various hosts and their associated vectors. These studies have documented many novel species in ticks and fleas collected from various domestic and wild animals such as *R. massiliae, R. slovaca, R. raoultii, R. africae*, and *Rickettsia* sp. R14 and in *R. felis*-like organisms such as *Rickettsia asembonensis*, *Candidatus* Rickettsia senegalensis, and *Rickettsia* spp. Genotype RF2125 (Table 4) [16,43,44,51,52,53,54,55].

### 3.2. Clinical Presentations

#### 3.2.1. Common Presentation

**Figure 2:**
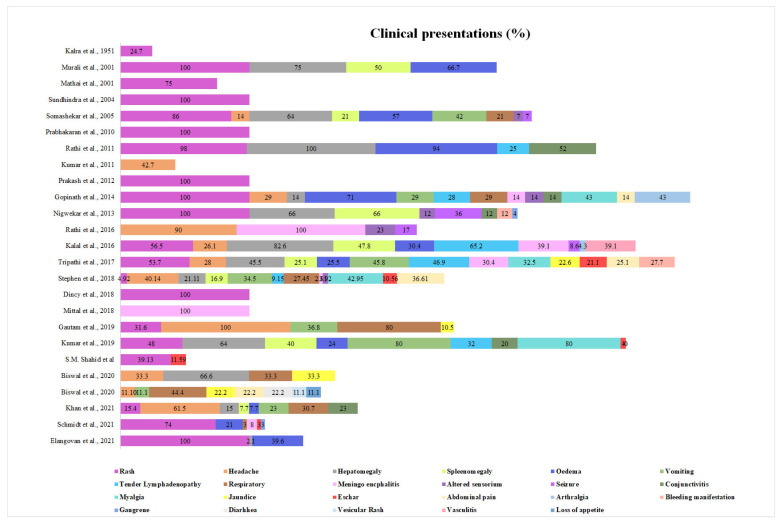
Clinical presentation among non-ST RDs in various studies. The figures are the proportion (%) of clinical presentation among the study population.

All prospective and most retrospective studies except case series or reports have focused on patients with AFI or PUO. The various clinical manifestations are listed in Figure 2. After fever, rash was the most common clinical manifestation associated with non-ST RDs. Many studies defined the study population with rash as inclusion criteria [11,12,15,34,45,66,67]. The prospective studies in febrile illness reported maculopapular rash in 4.9% to 98% [9,42]. The classical finding of eschar was not frequently reported in Indian studies, and it was reported with a range of 4% to 21.1% of the studied population [9,27,37,47]. Headache was reported in a range of 11% to 90% of the studied population [13,68]. An important clinical feature reported in many studies is facial edema or anasarca with a range of 24% to 94% [27,42]. The edema or anasarca were not clinically present in most of the cases, but marked congestion of most of the internal organs may be reported in fatal cases during autopsy [69]. Other clinical features were nausea or vomiting (11.1% to 80%) [13,26], respiratory symptoms (21% to 80%) [26,32], meningeal signs (2.8% to 39.1%) [9,35], altered sensorium (3.9% to 23%) [9,68], seizure (7% to 36%) [32,66], conjunctival effusion (12% to 52%) [42,66], myalgia (32.5% to 80%) [27,37], arthralgia (4.3% to 43%) [34,35], jaundice (10.5% to 33.3%) [26,49], abdominal pain (14% to 36.6%) [9,34], and bleeding manifestation (12% to 27.7%) [37,66]. Less common manifestations included vesicular rash (11.1%), vasculitis (39.1%), diarrhea (22.2%), and loss of appetite (11.1%) [13,35].

#### 3.2.2. Other Presentations

There were numerous case reports or case series which highlighted specific clinical presentations. The common presentations were ocular manifestations and/or neurological manifestations and purpura fulminans.

##### Ocular Manifestations

Apart from the common ocular manifestation, conjunctival effusion (12% to 52%) [42,66], post-febrile retinitis following the non-ST RDs was documented in many studies. A group of 12 patients over a period of three months presented with multifocal retinitis (mostly bilateral) with a history of fever approximately 4 weeks before the onset of defective vision. These patients were found to be positive for *R. conorii* infection (67% for non-ST RDs; 33.3% of each of SFG and TG rickettsioses). It was concluded that systematic fundus examination must be included in the routine evaluation of patients who present with fever or have a travel history of any endemic area of rickettsial pathogens. Ocular diagnosis should be one of the clinical diagnoses on encountering multifocal retinitis predominantly involving the posterior pole and macular involvement in the form of serous macular detachment or macular hard exudates [70]. Another retrospective study of 200 post-febrile retinitis cases documented 30% of SFG and 19.3% of TG infections using WFT [71]. A case of posterior focal retinitis in a cohort was found to be positive with non-ST RDs using WFT [72]. Few case reports of retinitis using WFT were documented [73,74]. A case of bilateral anterior uveitis with retinitis was documented with positive WFT for both OX-2 and OX-19 [75].

##### Neurological Manifestations

A retrospective study carried out at a children’s hospital from Akola, Maharashtra reported that 51 of 62 patients with rickettsial infections had symptomatic neurological involvement. It was observed that 21 patients had neurological manifestations as a main presenting feature. Various neurological manifestations observed included headache, meningeal signs, papilloedema, CSF and neuroimaging abnormalities, irritability, seizures, and focal neurological deficits [68]. Left-sided partial third cranial nerve palsy has been reported in Mumbai in a 25-year-old male as an accompanying clinical presentation of *Rickettsia* infection [76]. Few case reports of AES, meningoencephalitis, febrile delirium, and other neurological presentations were reported from various parts of the country [14,77,78].

##### Other Less Common/Atypical Presentations

Distinctive cutaneous eruptions were suggested to be an important clue in the early diagnosis of Indian tick typhus by a team of researchers in Bangalore. They studied 12 cases of rickettsial infections in which they observed that cutaneous eruptions developed almost 1 month before the development of fever. Erythematous lesions were observed in 11 cases and maculopapular lesions were observed in one case. Four cases were reported to have purpuric lesions as well. Instead of rapid eruptions, lesions were observed to appear daily until the patient was afebrile. The lesions were reported to subside in 7 to 10 days and were associated with post-inflammatory hyperpigmentation. Limbs were reportedly more involved than the trunk or face, and the sides of soles and instep of feet were found to be involved in all cases [79].

Three cases of purpura fulminans were reported from Karnataka to be positive for OX19, OX2, and OX19/OX2 using WFT [80]. Another single case was reported with positive WFT in different studies [81,82,83,84]. Cases of gangrene associated with non-ST RDs were reported [85,86]. Cases of malignant Mediterranean spotted fever were reported from New Delhi and Karnataka [87,88]. There were case reports of hepatitis and myocarditis associated with non-ST RDs [89,90].

#### 3.2.3. Laboratory Findings

Most of the studies have documented elevated liver enzymes (25.3% to 79.1% of cases) [9,37], leukocytosis (28% to 88.8%) [13,34], thrombocytopenia (28% to 100%) [34,49], and coagulopathy (14% in one study) [32].

#### 3.2.4. Treatment and Outcomes

Overwhelmingly, most of the studies documented that those patients administered doxycycline improved. One study in children showed 7.1% non-responsive to doxycycline therapy who improved with chloramphenicol [32]. The overall mortality associated with the non-ST RDs (SFG rickettsioses) ranged from 7.8% to 11.1%, and no mortality was documented among TG rickettsioses [13,37].

## 4. Discussion

As in the rest of the world [91], in India also, there has been an increase in reports regarding rickettsial pathogens. The true burden of the SFG and TG rickettsioses is still not well established, as most of the studies have used non-specific serological investigations such as the Weil–Felix test to differentiate among different rickettsial diseases. Most of these studies discussed the low sensitivity of the test is based on cases of scrub typhus rather than cases of spotted fever or typhus fever. [7,8,22,28]. Among the non-scrub typhus rickettsial infections, *R. conorii* was the most prevalent rickettsia pathogen, followed by *R. typhi*. Though few studies were conducted using IFA and MIF, these tests were not focused on various pathogenic rickettsia species and to limited species such *R. conorii* and *R. typhi* [10,11,31,32,36,40,42]. Because serological tests commonly used in these studies display substantial cross-reactivity between and within antigenic groups, it is difficult to conclude the true burden of SFG and TG rickettsioses. More PCR-based studies along with other serological tests, especially rapid point-of-care tests in appropriately defined clinical populations, might give more comparative value among these groups [12,43,44,47,48,50].

Application of molecular techniques has resulted in the discovery of at least a novel *Rickettsia* species closely related to *Candidatus* Rickettsia kellyi [45]. Similarly, many *Rickettsia* species have been detected in tick vectors in areas of endemicity of human infections [43,44,52,54,55]. Therefore, it is very possible that others might exist too, and will be picked up once specific diagnostics to the point of sequencing are carried out. Detailed studies on taxonomy using combination approaches such as MLST with other genes, MST (multispacer typing), mouse serotyping, and next-generation sequencing are also lacking from India [92]. Similarly, the role of any antigenic variation or diversity and phenotypic or genotypic variation in virulence and its role in variation in geographic differences need to be studied in the Indian population. In India, cases probably go undiagnosed as diagnostics for rickettsia are often missing for fever panels in India. Typical clinical presentation of RD is not always present; the disease is probably often underdiagnosed due to a lack of awareness [93].

There is emerging evidence for a new recognition of human rickettsioses in the Asia–Pacific region including Taiwan, Vietnam, Bhutan, and Malaysia [94,95,96,97]. Although there is increasing evidence of these infections in India, no broad studies have been conducted to determine the incidence per million people and the annual case fatality rate for these diseases. Therefore, the disease burden remains largely unknown in the Indian population.

Although the various vectors for rickettsia exist in India, there are no studies on their relative abundance through the seasons or any change in distribution in the recent past to correlate with the increase in the incidence of human rickettsial infections. The role of different vectors and their distribution, biology, and life cycles—as well as seasonal variations with tick activity and their association with the incidence of cases and distribution in rural, semi-urban, and urban areas—need to be explored further. Similarly, studies on seroprevalence in domestic and wild animals are limited in India, which could yield important epidemiological information on these diseases.

Finally, there are no national-level reference laboratories in India with cell culture facilities for the isolation of rickettsia. The establishment of such a reference laboratory would be helpful to study these pathogens in detail. Moreover, there is no reporting system such as the CDC in the US for rickettsial infections. This system, with the help of a reference laboratory, would provide accurate estimates of the disease burden as well as address the gaps outlined above.

## 5. Conclusions

To conclude, rickettsial infections occur in India but are probably underdiagnosed due to a lack of awareness. Clinicians and microbiologists should be made aware of the epidemiology and diagnostic tests for them. A single serum sample at the time of presentation might not be adequate, so paired (acute and convalescent) sera for IFA, considered to be the gold standard serology, is required. Better still, molecular testing using the panel of PCRs is recommended for speciation along with other serological investigations for confirmation. Sequencing to determine novel rickettsial pathogens should be attempted at laboratories with the facilities to accurately map the epidemiology of these infections.

## Figures and Tables

**Table 1:** Prevalence of non-ST RDs based on the Weil–Felix test.

Author	Region	Study Details	RD (%)	SFGR (%)	TGR (%)	Undifferentiated Rickettsioses (%)
Kamarasu et al., 2007 [7]	South India (Tamil Nadu)	964 prospective samples over 2 years in various PHCs	4.6	ND	ND	ND
Prabhakaran et al., 2010 [15]	South India (Andhra Pradesh)	39 prospective patients of fever with rash	7.7	0	0	7.7
Vinoth et al., 2011 [16]	South India (Karnataka)	200 suspected outbreak patients	9	4	0	5
Mittal et al., 2012 [17]	North India (New Delhi)	824 prospective samples [737 (initial 5 years) and 87 in subsequent 5 years]	5.2	3.9	0.8	0.5
Udayan et al., 2014 [18]	South India (Karnataka)	100 prospective febrile patients	55	45	10	4
Danave et al., 2015 [19]	West India (Maharashtra)	156 prospective febrile patients	15	3.8	0	11.5
Kumar et al., 2015 [20]	South India (Karnataka)	60 prospective PUO patients	5	1.7	1.7	1.7
Rashmi et al., 2015 [21]	South India (Karnataka)	133 prospective AUFI patients	44.8	10.3	6.8	27.6
Thomas et al., 2016 [22]	South India (Karnataka, Andhra Pradesh and Tamil Nadu)	262 children of suspected rickettsial infection (Retrospective)	20	ND	ND	ND
Narvencar et al., 2017 [23]	West India (Goa)	55 prospective AUFI patients	20	16.4	3.6	1.8
Sanap et al., 2017 [24]	West India (Maharashtra)	1464 prospective PUO patients	10.1	4.6	3.5	2.1
Goyal et al., 2018 [25]	North India (New Delhi)	22 prospective PUO patients	41	27.3	13.6	13.6
Gautam et al., 2019 [26]	North India (New Delhi)	370 prospective AUFI patients	5.1	3.8	1.4	ND
Kumar et al., 2019 [27]	South India (Karnataka)	324 prospective samples from children with fever	9.2	1.5	7.7	1.5
Akshatha et al., 2020 [28]	South India (Andhra Pradesh)	120 prospective samples from febrile patients	21	ND	ND	20.8
Kavirayani et al., 2021 [8]	South India (Karnataka)	214 AFI patients	7.1	ND	ND	ND

AUFI—acute undifferentiated febrile illness; PUO—pyrexia of unknown origin; AFI—acute febrile illness; SFGR—spotted fever group rickettsioses; TGR—typhus group rickettsioses; PHCs—primary health centers; ND—no details.

**Table 2:** Prevalence of non-ST RDs based on various serological tests.

Author	Region	Study Details	Diagnostic Methods Used	Results
Cross-sectional studies				
Kalra et al., 1951 [3]	North India (Kashmir)	178 endemic patients	53 patients tested for WFT and CFT	WFT = TGR—90.6% (47); SFGR—1.9% (1) CFT = Murine typhus—70% (37); 11—Epidemic typhus—20.8% (11); 5—non reactive
Mane et al., 2019 [29]	Central India (Gorakhpur, Uttar Pradesh)	329 AFI patients (294 samples)	*R. conorii* IgG/IgM ELISA kit *R. typhi* IgG/IgM ELISA	SFGR = IgM—13.6% (40)/IgG—36.7% (108); TGR = IgM—7.1% (21), IgG—15.3% (45); undifferentiated rickettsioses = 1.4% (4) (total 218 case)
Devamani et al., 2020 [30]	South India (Tamil Nadu)	1353 samples	IgG ELISA for SFG/TG	IgG ELISA—15.8% (10.4% SFGR and 5.4% TGR rickettsioses)
Khan et al., 2021 [6]	Northeast India (Assam, Meghalaya, Tripura)	2360 samples; (772—Assam 768—Meghalaya 820—Tripura)	IgG ELISA for SFG/TG	Overall—1.8% (1.4% SFGR and 0.3% TGR) Assam—1.4% (1.2% SFGR and 0.2% TGR) Meghalaya—0.5% (0.3% SFGR and 0.2% TGR) Tripura—2.9% (2.4% SFGR and 0.5% TGR)
Prospective studies (In defined population)				
Murali et al., 2001 [11]	South India (Tamil Nadu)	57 children of fever with rash	WFT, immuno haemagglutination (IHA; in 4 patients)	WFT = 21% (12); SFGR—7% (4), TGR—10.5% (6), undifferentiated rickettsioses—3.5% (2); IHA = SFGR—3/4, TGR—1/4)
Mathai et al., 2001 [31]	South India (Tamil Nadu)	475 PUO patients	WFT, indirect haemagglutination assay (RBC sensitised with *R. typhi, R. rickettsii* antigens)	SFGR—1.3% (6), TGR—1.3% (6); (Total—2.6%)
Somashekar et al., 2005 [32]	South India (Tamil Nadu)	180 children with PUO	WFT, ELISA or microIFA	43 patients of RDs; 15 non scrub typhus patients; TGR—1.1% (2), SFGR—7.8% (14)
Kulkarni et al., 2009 [10]	West India (Maharashtra)	156 PICU patients suspected with RDs	WFT, SFGR ELISA in 26 patients, IFA in CDC for 2 patients	WFT positive in 73 patients and SFGR was predominant; ELISA = SFGR—13.5% (80.8%; 21/26); IFA = 2 patients *R. conorii*
Chrispal et al., 2010 [33]	South India (Tamil Nadu)	398 AFI in patients	SF IgM ELISA	ELISA = SFGR—1.8% (7)
Gopinath et al., 2014 [34]	South India (Vellore, Tamil Nadu)	398 AUFI in adult with SFGR defined patients	SF IgM ELISA	ELISA = SFGR—1.76% (7)
Kalal et al., 2016 [35]	South India (Karnataka, Andhra Pradesh and Tamil Nadu)	103 children with suspected Rickettsial disease	ELISA (*R. conorii* IgM/IgG kit), WFT	ELISA = SFGR—37.1% (23); WFT = 44.4% (4) of ELISA positive patients
Koralur et al., 2016 [36]	South India (Karnataka)	1036 patients with suspected RDs	WFT, IFA	IFA= SFGR—2.1% (22); TGR—1.8% (19) (Cross reactions; 18 patients were positive for *Rickettsia australis*, 16 for *Rickettsia honei*, 15 for *R. conorii*, 16 for *Rickettsia africae*, 15 for *Rickettsia rickettsii*, 11 for *Rickettsia felis*, 4 for *Rickettsia prowazekii* and 6 patients for *R. typhi*)
Tripathi et al., 2017 [37]	Central India (Uttar Pradesh)	432 PUO patients	WFT, ELISA for specific IgM antibody against *R. conorii* and IFA for IgM antibody against *R. conorii/R. typhi*	WFT = 46.3% (200); ELISA and IFA = 26.6% (115)
Stephen et al., 2018 [9]	South India (Puducherry)	320 clinically defined patients for RDs	WFT, SFG specific *R. conorii* IgM/IgG ELISA	21.3% (68 patients) by WFT; 11.6% (37) by ELISA; 142 SFGR by all test (44.4%);
Shriharsha et al., 2019 [38]	South India (Karnataka)	231 AFI patients	WFT, *R. conorii* IgG/IgM ELISA	WFT = 36.3% SFGR; 2.1% TGR; 6.9% undifferentiated rickettsioses IgG ELISA—11.7% SFGR
Khan et al., 2021 [39]	Central India (Gorakhpur, Uttar Pradesh)	217 children with AFI	IgG ELISA for SFGR/TGR	IgG ELISA—6% SFGR (13 children) and 0% TGR
Mansoor et al., 2021 [40]	North India (Srinagar)	344 AUFI patients	WFT, ELISA, IFA	WFT = 3.5% (12) non-scrub typhus RDs patients (2.3% SFGR; 1.2% TGR); IgM/IgG TGR ELISA—8.1% (28/344); IgM/IgG SFGR ELISA—10.5% (36/344); IFA—8.2% (4.7% SFGR and 3.5% TGR)
Schmidt et al., 2021 [41]	South India (Vellore, Tamil Nadu)	77 AUFI patients	IgM/IgG ELISA for SFGR	All 77 IgG/IgM SFGR positive cases enrolled- IgM/IgG response over time
Retrospective studies (In defined population)				
Rathi et al., 2011 [42]	West India (Maharashtra)	161 children with RD defined patients	SFGR IgM ELISA, WFT, IFA IgM for SFGR	52 patients SFGR (70%) among 75 RDs ELISA = SFGR—32.3% (47) (10 of ELISA positive were tested for IFA—all positive)

AUFI—acute undifferentiated febrile illness; PUO—pyrexia of unknown origin; AFI—acute febrile illness; PICU—pediatric intensive care unit; SFGR—spotted fever group rickettsioses; TGR—typhus group rickettsioses; WFT—Weil–Felix test; ELISA—enzyme-linked immunosorbent assay; CFT—complement fixation test; IFA—indirect immunofluorescent assay.

**Table 3:** Prospective studies with molecular evidence for non-ST RDs in India.

Author	Region	Total Study Population	Diagnostic Methods Used	Results	Most Closely Related Pathogens based on Submitted Sequence in NCBI GenBank.
Prakash et al., 2012 [12]	South India (Vellore, Tamil Nadu)	58 patients of suspected SFGR fever with rash sample	IgM ELISA in serum, nPCR (targeted *glt*A, *omp*A, *omp*B, 17*k*Da genes) in skin biopsy	34 cases PCR confirmed (58.6%), 27/34—ELISA positive	*Rickettsia parkeri*, *Rickettsia africae, Rickettsia sibirica*, *Rickettsia mongolotimonae*, *Rickettsia japonica*, *Rickettsia honei, Rickettsia rickettsii*, *R. conorii*, *Rickettsia* spp. IG-1, *Candidatus* Rickettsia kellyi, *Rickettsia slovaca*
Chahota et al., 2015 [43]	North India (Himachal Pradesh)	Clusters of 300 fever patients from 3 villages	WFT, PCR (targeted *glt*A and *omp*B gene) in blood	7 WFT positive cases tested (2.3) with PCR which were negative	No PCR positive
Khan et al., 2016 [44]	Northeast India (Assam, Arunachal Pradesh and Nagaland)	1265 random samples of residents of scrub typhus endemic region.	indirect ELISA, PCR (targeted *glt*A and *omp*B gene) in seropositive serum samples	SFGR—175 (13.8%) TGR—53 (4.2%) PCR—nil	No PCR positive
Dincy et al., 2018 [45]	South India (Vellore, Tamil Nadu)	30 of 35 clinically defined patients	PCR (targeted *glt*A, *omp*A, *omp*B, 17*k*Da genes) in biopsy from rash and ELISA in serum, HPE of skin biopsy of rashes	30 cases PCR and /or ELISA	No sequence data available
Mittal et al., 2018 [46]	Central India (Gorakhpur, Uttar Pradesh)	389 AES patients	PCR for SFGR in CSF and/or Serum sample (targeted IGS 23S-5S region), in brain biopsy sample (targeting *omp*A)	4 (1%) from CSF or Serum, 1 positive from brain biopsy	No sequence data available
Shahid et al., 2019 [47]	South India (Karnataka)	262 AFI blood samples	WFT, PCR (targeted *glt*A gene) in whole blood	WFT—116 (44.3%), *glt*A PCR—69/116 cases (59.5%)	No sequence data available
Khan et al., 2019 [48]	Northeast India (Assam, Arunachal Pradesh, Nagaland, Manipur, Mizoram, Meghalaya and Tripura)	2199 (762 AES patients; 1437 PUO patients)	ELISA (Antibody against TGR), snPCR (targeted 17*k*Da gene) in serum samples	ELISA for TGR: 30/762; 3.9% among AES, 39/1437; 2.7% among PUO (Assam—0, Arunachal Pradesh—0, Nagaland—2.2%, Manipur—3.8%, Mizoram—2%, Meghalaya—0 and Tripura—6.3%) PCR—No positive among 15 ELISA positive sample	No PCR positive
Biswal et al., 2020 [49]	North India (Chandigarh)	51 patients of PUO	nPCR (targeted *omp*A gene) in clot sample	3 cases of *R. conorii* (5.8%)	*R. conorii* clone 09 (KR401144) and *R. conorii subsp. conorii* clone 45 (JN182802)
Biswal et al., 2020 [13]	North India (Chandigarh)	200 patients of AUFI	nPCR (targeted *glt*A gene) in clot sample	7% (14 cases), SFGR -4%, TGR-3%	*Rickettsia conorii* and *Rickettsia typhi*
Elangovan et al., 2021 [50]	South India (Vellore, Tamil Nadu)	175 patients of suspected SFGR with fever and rash	IgM ELISA in serum, nPCR (targeted *glt*A, *omp*A genes) in buffy coat	50% (48 SFGR cases; 48/96 by either by PCR or ELISA; 22%—21/96 by PCR; 28%—27/96 by ELISA)	Uncultured *Rickettsia* sp. Clone cmc 08 (GQ260637), *Rickettsia* sp. Tenjiku01 (LC089864), *Rickettsia raoulti* (KR131756) and *Rickettsia parkeri* (CP040325)

AUFI—acute undifferentiated febrile illness; PUO—pyrexia of unknown origin; AES—acute encephalitis syndrome, AFI—acute febrile illness; SFGR—spotted fever group rickettsioses; TGR—typhus group rickettsioses; WFT—Weil–Felix test; PCR- polymerase chain reaction; nPCR—nested polymerase chain reaction; ELISA—enzyme-linked immunosorbent assay.

**Table 4:** Entomological studies on vectors of non-ST RDs in India.

Author	Region	Host	Vector	*Rickettsiae*
Kumar et al., 2011 [51]	North India (Kangra)	Domestic ruminants, Rodents	Ticks (*Ixodes ricinus*, *Rhipicephalus sanguenieussanguineus)*	ND
Vinoth et al., 2011 [16]	South India (Kolar)	Rodents	ND	1 *Proteus* OX-2 positive
Chahota et al., 2015 [43]	North India (Himalayan Region)	Rodents	Rat flea (*Ceratophyllus fasciatus)*	*Rickettsia* sp. R14
Hii et al., 2015 [52]	West India (Mumbai), North India (Delhi & Rajasthan)	Stray dogs	Cat flea (*Ctenocephalides felis Orientis*—89.6%, *Ctenocephalides felis felis*—10.4%)	*Rickettsia* sp. genotype RF2125
Khan et al., 2016 [44]	Northeast India (Assam)	Dog, cattle, cats	Cat fleas *(Ctenocephalides felis)*	*Candidatus* Rickettsia senegalensis
Bhuyan et al., 2016 [53]	South India (Nilgiris, Tamil Nadu)	Domestic and peri-domestic rats	*Ornithonyssus bacoti*	ND
Nimisha et al., 2019 [54]	South India (Kerala)	Domestic ruminants (cattle and goats) and wild animals (sambar deer and elephant calf)	*Rhipicephalus haemaphysaloides*, *Haemaphysalis bispinosa*, *Amblyomma* sp.	*Rickettsia massiliae, Rickettsia slovaca, Rickettsia raoultii, Rickettsia africae*
Nataraj et al., 2020 [55]	South India (Puducherry)	Pets (dogs) and domestic ruminants (buffaloes, cattle, and goats)	Cat fleas *(Ctenocephalides felis felis)*	*Rickettsia asembonensis*

## Data Availability

Data sharing not applicable. No new data were created or analyzed in this study. Data sharing is not applicable to this article.
